# A100 ADULT GASTROENTEROLOGIST PERSPECTIVES ON AN EDUCATIONAL PLATFORM TO SUPPORT YOUNG ADULTS WITH INFLAMMATORY BOWEL DISEASE

**DOI:** 10.1093/jcag/gwae059.100

**Published:** 2025-02-10

**Authors:** Z Mohmand, A Miatello, B Allemang2, A Tausif, S Lawrence, C Deslandres, C.Education Committee, Y Wang, J de Guzman, K Lee, M Barwick, E I Benchimol, N Fu, N Bollegala

**Affiliations:** Queen’s University, Kingston, ON, Canada; SickKids Research Institute, Toronto, ON, Canada; SickKids Research Institute, Toronto, ON, Canada; Crohn’s and Colitis Canada, Toronto, ON, Canada; BC Children’s Hospital, Vancouver, BC, Canada; Centre Hospitalier Universitaire Sainte-Justine, Montreal, QC, Canada; Canadian Children’s IBD Network, Toronto, ON, Canada; The University of Melbourne Melbourne Medical School, Melbourne, Victoria, Australia; Crohn’s and Colitis Canada, Toronto, ON, Canada; Crohn’s and Colitis Canada, Toronto, ON, Canada; SickKids Research Institute, Toronto, ON, Canada; The Hospital for Sick Children, Toronto, ON, Canada; Richmond Hospital, Richmond, BC, Canada; Women’s College Hospital, Toronto, ON, Canada

## Abstract

**Background:**

Adolescents and young adults (AYAs) with inflammatory bowel disease (IBD) encounter challenges during the transition from pediatric to adult healthcare, where they must manage their care with increasing independence. The Transition Adolescent & Young Adults implementioN (IBD-TrAYN) is a randomized clinical trial (NCT05221281) of a program to address care gaps during this critical period. As part of the intervention arm, we developed an online educational platform with 19 modules to enhance disease knowledge and self-management skills among AYAs.

**Aims:**

This quality improvement project aims to understand the perspectives of adult gastroenterologists (GIs) on the relevance of the module content for AYAs with IBD.

**Methods:**

Adult GIs with experience treating AYAs (18-25y) with IBD in Canada were recruited using purposive and snowball sampling techniques. Three virtual focus groups were conducted to explore perceptions of module applicability and potential gaps in the curriculum. Content analysis was performed on the transcripts for emergent themes by two independent reviewers.

**Results:**

Eleven GIs were recruited, who recognized the educational platform as a valuable tool for addressing critical gaps in care due to limited clinic time, allied health support, and varying patient knowledge and transition readiness levels. Modules identified as high-priority included disease self-management, mental health, and sexual health. Concerns about information overload led to suggestions for combining related topics, such as family planning and sexual health, and integrating features like searchability and interactive elements to make the content more engaging and personalized. Participants recommended the development of education modules to support parallel caregiver role transitions. Participants stressed the importance of a flexible, accessible approach that allows AYAs access to relevant information at their own pace.

**Conclusions:**

Adult GIs endorsed the value of the platform and its relevance in addressing their clinical priorities and the needs of their AYA patients. The perspectives gathered will inform content refinement to prevent information overload and enhance the curriculum, ultimately contributing to enhanced knowledge and transition readiness for AYAs with IBD.

**Funding Agencies:**

CCC

